# Heterogeneous Intracellular Trafficking Dynamics of Brain-Derived Neurotrophic Factor Complexes in the Neuronal Soma Revealed by Single Quantum Dot Tracking

**DOI:** 10.1371/journal.pone.0095113

**Published:** 2014-04-14

**Authors:** Anke Vermehren-Schmaedick, Wesley Krueger, Thomas Jacob, Damien Ramunno-Johnson, Agnieszka Balkowiec, Keith A. Lidke, Tania Q. Vu

**Affiliations:** 1 Department of Biomedical Engineering and Center for Spatial Systems Biomedicine, Oregon Health & Science University, Portland, Oregon, United States of America; 2 Department of Physics & Astronomy, University of New Mexico, Albuquerque, New Mexico, United States of America; 3 Department of Integrative Biosciences, Oregon Health & Science University, Portland, Oregon, United States of America; “Mario Negri” Institute for Pharmacological Research, Italy

## Abstract

Accumulating evidence underscores the importance of ligand-receptor dynamics in shaping cellular signaling. In the nervous system, growth factor-activated Trk receptor trafficking serves to convey biochemical signaling that underlies fundamental neural functions. Focus has been placed on axonal trafficking but little is known about growth factor-activated Trk dynamics in the neuronal soma, particularly at the molecular scale, due in large part to technical hurdles in observing individual growth factor-Trk complexes for long periods of time inside live cells. Quantum dots (QDs) are intensely fluorescent nanoparticles that have been used to study the dynamics of ligand-receptor complexes at the plasma membrane but the value of QDs for investigating ligand-receptor intracellular dynamics has not been well exploited. The current study establishes that QD conjugated brain-derived neurotrophic factor (QD-BDNF) binds to TrkB receptors with high specificity, activates TrkB downstream signaling, and allows single QD tracking capability for long recording durations deep within the soma of live neurons. QD-BDNF complexes undergo internalization, recycling, and intracellular trafficking in the neuronal soma. These trafficking events exhibit little time-synchrony and diverse heterogeneity in underlying dynamics that include phases of sustained rapid motor transport without pause as well as immobility of surprisingly long-lasting duration (several minutes). Moreover, the trajectories formed by dynamic individual BDNF complexes show no apparent end destination; BDNF complexes can be found meandering over long distances of several microns throughout the expanse of the neuronal soma in a circuitous fashion. The complex, heterogeneous nature of neuronal soma trafficking dynamics contrasts the reported linear nature of axonal transport data and calls for models that surpass our generally limited notions of nuclear-directed transport in the soma. QD-ligand probes are poised to provide understanding of how the molecular mechanisms underlying intracellular ligand-receptor trafficking shape cell signaling under conditions of both healthy and dysfunctional neurological disease models.

## Introduction

The dynamic trafficking of activated ligand-receptor complexes is believed to play a key role in the spatiotemporal regulation of cell signaling [1]–[3]. In neurons, growth factor ligands bind to Trk tyrosine kinase receptors forming a signaling complex that is internalized and trafficked within the neuronal intracellular environment to shape essential neuronal signaling functions [4]–[7]. While the molecular identities of signaling are increasingly well- delineated, the real-time molecular dynamics of growth factor complex trafficking within cells are not well studied or understood. Focus has been placed on axonal trafficking [8]–[11], but little is known about growth factor-activated Trk dynamics in the neuronal soma, particularly at the molecular scale, due in large part to technical challenges in tracking individual ligand-receptor complexes for extended periods of time and over long distances (several microns) within neurons.

Brain-derived neurotrophic factor (BDNF) binding to TrkB tyrosine kinase receptors leads to dimerization and autophosphorylation of Trk receptors that are followed by the internalization of activated BDNF-TrkB complexes into the cytoplasm [12]–[15]. Internalized BDNF-TrkB complexes are believed to continue to initiate MAPK and PI3K downstream signaling that controls a host of critical functions including neuronal survival, development, learning and memory, pain processing, and afferent cardiorespiratory control [6], [16]–[24]. BDNF-TrkB trafficking is essential to major neuronal functions such as dendritic branching [15] and activity-dependent synaptic plasticity [25]. Moreover, BDNF signaling is implicated in important neurodegenerative disorders (e.g. Alzheimer's disease and Huntington's disease), as well as psychiatric disorders (e.g. major depression) [26], [27]. Specifically, BDNF-TrkB trafficking is becoming increasingly associated with devastating neurodegenerative and developmental disorders [11], [14], [28], [29]. While pioneering studies have focused on the linear transport of growth factor complexes along axonal and dendritic processes [4], [7], little is known about the molecular (nanometer scale) dynamics of BDNF-TrkB and other growth factors within the soma of neurons, which is postulated to differ from that at neuronal processes [5], [7], [30]. Information on BDNF-TrkB dynamics, particularly in the neuronal soma, is fundamental to elucidating the interface between growth factor trafficking and signaling.

Quantum dots (QDs) are intensely bright and photostable fluorescent nanoparticles that have proven invaluable in characterizing the dynamics of single ligand-receptor complexes on the extracellular plasma membrane surface of live cells [31]–[36]. The use of QDs in investigating the dynamics of activated ligand-receptor complexes following their internalization from the plasma membrane, however, has yet to be fully exploited. We and others have demonstrated the use of QDs for studying growth factor and other neural receptor trafficking in the neuronal cytoplasmic environment [36]–[41], and the use of QDs for the general study of intracellular protein dynamics is emerging [42]. However, examples of QD probe applications that shed new dynamic information on specific intracellular signaling proteins are currently scant, characterization of QD-conjugated brain-derived neurotrophic factor (QD-BDNF) probe interactions with TrkB have not yet been reported in detail, and more importantly, the molecular scale dynamics of individual growth factor-Trk complexes are not well described in neuronal soma, using QDs or any other tools.

Here, we establish that QD-BDNF binds to TrkB receptors with high specificity, activates TrkB downstream signaling, and exhibits long-term tracking capability within the soma of live neurons. The important contribution of QD-BDNF probes to studying intracellular trafficking is demonstrated by the valuable new insight into the dynamics of individual BDNF complexes measured in nodose ganglion (NG) sensory neurons which employ BDNF-TrkB signaling in a retrograde [43], and autocrine [44] manner. Real-time measurements show that, following activation, one can observe individual BDNF complexes undergoing internalization, recycling, and intracellular transport events. Interestingly, such dynamic events show little synchronicity of occurrence and instead possess widely heterogeneous underlying dynamics, including extended durations of sustained rapid transport as well as immobility (30–120 s). Moreover, the path trajectories formed by dynamic individual BDNF complexes show no apparent end destination as BDNF complexes can be found meandering along circuitous paths several microns deep throughout the neuronal soma. The rich diversity of BDNF dynamics revealed by QD-ligand intracellular probes contrasts previously reported linear axonal transport data and make clear the urgent need for models of ligand-receptor intracellular dynamics that surpass our current notion of primarily nuclear-directed, somatic transport. QD-ligand probes are poised not only to identify the specific molecular mechanisms that underlie the rich diversity of the intracellular ligand-receptor trafficking dynamics that we report here, but also to address how such dynamics serve to shape cell signaling.

## Materials and Methods

### Ethics Statement

All procedures were approved by the Institutional Animal Care and Use Committee of the Oregon Health & Science University (Protocol Number: IS00001990), and conformed to the *Policies on the Use of Animals and Humans in Neuroscience Research* approved by the Society for Neuroscience and the *NIH Guide for the Health and Use of Laboratory Animals*.

### Generation of QD-BDNF probes

QD-BDNF was produced by high-affinity binding of biotinylated BDNF to streptavidin-QDs. BDNF (20 µg) was biotinylated via covalent linkage of biotin (EZ-NHS PEG_4_ biotin kit, Pierce, Rockford, IL; Cat# 21329) to the lysine and N-terminal amino groups of recombinant human BDNF (Pepro-Tech, Rocky Hill NJ; Cat# AF450-02) following the manufacturer's instructions. Biotinylated-BDNF product was purified and concentrated by centrifugation (Amicon Ultra-0.5 mL 3 kD Centrifugal Filters, EMD Millipore, Billerica, MA), aliquoted, and stored at −20°C. Quantum dot-BDNF (QD-BDNF) probes were made fresh by incubating biotinylated BDNF with streptavidin-QD_655_ or QD_625_ (Life Technologies, Carlsbad CA; Cat#s Q10121MP and Q22063 respectively) at a molar ratio of 1∶1 [39], [45]. Synthesized QD-BDNF probes were imaged on glass coverslips and fluorescence intensity profiles showed that most QD-BDNF fluorescent puncta were comprised of square pulse-shaped on-off states (90.1%, n = 22), indicating that QD-BDNF probes were composed of single QDs. Streptavidin-QDs, unmodified BDNF and Alexa_488_-BDNF (Ax-BDNF) were used as controls. Ax-BDNF probes were made by incubating biotinylated-BDNF with streptavidin-conjugated Alexa Fluor_488_ (Life Technologies; Cat# S11223).

### Plate assay of BDNF biotinylation

Indirect ELISA assays were used to characterize the degree of biotinylation of BDNF. All ELISA reactions were performed at room temperature unless otherwise noted. Briefly, 96-well plates (NUNC, Thermo Scientific, Rochester, NY) were coated with 75 ng/ml anti-human BDNF antibodies (R&D systems, Minneapolis, MN; Cat# MAB848) in 25 mM carbonate buffer pH 9.7 overnight at 4°C. Next, plates were blocked with ELISA blocking solution (Pierce; Cat# N502) for 1 h, incubated with either biotinylated or unmodified BDNF at different concentrations (0.625–40 ng/ml) for 2 h, followed by avidin-HRP (Pierce; Cat# N100) at 1∶10000 dilution for 1 h, and developed with K-blue MAX TMB substrate (Tetramethylbenzidine; Neogen, Lansing, MI). The reaction was stopped with 1 N HCl, and absorbance was measured at 450 nm using a microplate reader (Infinite 200 PRO, Tecan, Männerdorf, Switzerland).

### Plate assays of biotin-BDNF and QD-BDNF binding to TrkB

BDNF ELISAs were used to determine if biotin interfered with the ability of BDNF to bind to the TrkB receptor (BDNF Emax-ImmunoAssay System, Promega, Madison, WI). Assays were performed according to the manufacturer's instructions, with the following modification: 96-well plates were coated with TrkB (R&D systems; Cat# 688-TK; 15–1000 ng/ml) instead of the monoclonal anti-BDNF antibody. Blocked plates were incubated with biotinylated-BDNF or unmodified-BDNF (100 ng/ml (3.4 nM), 1 h) followed by anti-BDNF antibodies and secondary antibodies coupled to HRP, and developed with TMB. Separate TrkB receptor-coated ELISA plate assays were used to compare the TrkB binding to unmodified BDNF, QD-BDNF, and control streptavidin-QD (100 ng/ml), and incubated in the dark (1 h). QD_655_ fluorescence was measured (Excitation 340 nm, Emission 655 nm) using an Infinite 200 PRO microplate reader.

### Animals

Embryos (day 16.5) and postnatal day (P) 1 pups were obtained from time pregnant Sprague Dawley rats (Charles River Laboratories, Wilmington, MA).

### Cell Cultures

All sister cultures were grown in a 37°C, humidified 5% CO_2_ environment. *Embryonic neuronal cultures*: E16.5 nodose ganglion (NG) cultures were prepared as described previously [46] with some modifications. Pregnant female rats were euthanized by exposure to CO_2_ followed by an intraperitoneal injection of Euthasol (0.1 mg/g). Embryos were excised in 0.1 M PBS, NGs were removed and collected in ice-cold Ca^2+^/Mg^2+^-free Dulbecco's phosphate-buffered salt solution (Mediatech, Herndon, VA), digested in 1 mg/ml dispase in DMEM (1 h, 37°C) (Stemcell, Vancouver BC, Canada; Cat# 07923), followed by trituration in Neurobasal medium supplemented with B-27 serum-free supplement (Life Technologies), 0.5 mM L-glutamine, 2.5% fetal bovine serum (HyClone, Logan, UT), 1% Penicillin-Streptomycin-Neomycin antibiotic mixture (Life Technologies). Dissociated neurons (1×10^3^/well) were center-plated on glass coverslips (8 mm), pre-coated with 0.1 mg/ml poly-D-Lysine (PDL) and 0.1 µg/ml laminin. Cultures were grown in Neurobasal medium for 3 days in the presence or absence of BDNF (see “Embryonic neuronal survival”). E18 rat cortical cultures were prepared as described previously [47]. Briefly, cortices were removed and collected in ice-cold Ca^2+^/Mg^2+^-free Dulbecco's phosphate buffered salt solution (Mediatech), enzymatically digested in 0.1% crystallized trypsin-3X (Worthington Biochemical Corp., Lakewood, NJ) for 1 h at 37°C, followed by trituration in Neurobasal medium (Life Technologies) as described above, and grown in Neurobasal medium for 6-7 days. *Postnatal neuronal cultures*: P1-2 NG cultures were prepared as previously described [48] with an added step of Percoll density gradient for neuronal enrichment: following the enzymatic digestions, the dissociated NG cells were put on a 35%-60% Percoll gradient and centrifuged (10 min, 4°C, 800 g), and washed twice with Neurobasal A medium (supplemented as the Neurobasal medium, see “embryonic neuronal cultures’). Neuron-enriched NG dissociates were center-plated (2 NGs/well) on 0.1 mg/ml PDL and 0.1 µg/ml laminin-coated cover slips (18 mm). Cultures were grown in Neurobasal-A medium for 4–5 days. Cytosine β-D-arabinofuranoside (AraC, 10 µm, Sigma) was added to the culture medium after 24 hours. *N2A neuroblastoma cell line cultures*: Neuro 2A neuroblastoma cells (ATCC-CCL-131, Manassas, VA) were prepared as described [36]. Neurons were center-plated on 0.1 mg/ml PDL–coated coverslips (18 mm) and grown in a medium composed of 47.5% D-MEM, 47.5% Opti-MEM, and 5% FBS.

### Fluorescent assays of QD-BDNF binding in neuronal cell lysates

Neuronal cultures (NG, cortical and N2A) were grown in 6 well plates for 4–5 days (2.5×10^4^, 1×10^5^ and 1×10^5^, respectively). Following incubations with QD_655_-BDNF or streptavidin-QD_655_ (15 min), the cells were either imaged or lysed (20 mM Tris base, 20 mM Tris HCl, 137 mM NaCl, 1% nonidet-P40, 10% glycerol, 1 mM PMSF, 0.5 mM sodium vanadate, pH 8.0). QD_655_ fluorescence of cell lysates was measured (Excitation 340 nm, Emission 655 nm) using an Infinite 200 PRO microplate reader.

### Embryonic neuronal survival assays of QD-BDNF activity

E16.5 NG cultures were grown for 3 days in Neurobasal medium in the absence or presence of BDNF (1, 2 or 4 nM BDNF), streptavidin-QDs (4 nM) and QD-BDNF (4 nM), rinsed in PBS and fixed (4% paraformaldehyde, 30 min, RT), rinsed in PBS, and processed for neurofilament (NF)-immunocytochemical staining. For QD-BDNF receptor binding specificity, E16.5 NG cultures were pre-incubated with anti-TrkB function blocking antibodies (BD Transduction, San Jose, CA; Cat# 610102) for 1 h before adding the BDNF and QD-BDNF. To label neurons, we used anti- NF-68 and NF-160 antibodies (Sigma; Cat# N5139 and N5264) as previously described [48]. Following staining, cultures were mounted with ProLong-Gold (Life Technologies) and imaged (Excitation 550 nm/Emission 570 nm). The number of NF-immunoreactive neurons in each culture was counted and expressed as number of neurons per 10 mm^2^ area.

### QD-BDNF treatment and Alexa-WGA membrane labeling of cells

Neuronal cultures (5 days *in vitro*) were pre-incubated in Neurobasal (embryonic) or Neurobasal A (postnatal) medium without B27 supplement (30 min, 37°C) and treated with QD-BDNF or control streptavidin-QDs. Neurons were incubated at 37°C with QD_655_-BDNF probes (25–400 pM), fixed in 4% PFA (20 min, RT), rinsed in PBS, and placed in borate buffer (10 mM, pH 8.0) for immediate imaging or subsequent antibody staining. For QD-BDNF trafficking in live cells, neurons were rinsed with PBS and placed in plating medium without B27 and supplemented with 50 ng/ml Vitamin C and 25 mM HEPES (30 min), incubated with QD_625_-BDNF for 1–2 min, rinsed and imaged. In both cases, and at 30 s prior to the end of BDNF treatment, Alexa Fluor_488_-WGA (2 µg/ml, Life Tech, Cat# W11261) was added to label the plasma membrane. NG neurons showed heterogeneity of QD-BDNF binding (n = 100): 13.8% showed no QD-BDNF binding, 34.8% showed QD-BDNF binding but no internalization, and 51.5% showed QD-BDNF binding and internalization. For the purposes of studying BDNF internalization and trafficking, we studied only the population of NG neurons that showed QD-BDNF internalization.

### Blinking analysis

To examine the blinking properties of the QD-BDNF probes, we imaged the probes on a glass coverslips and inside neurons (27 frames per second). Profiles of total intensity of individual QD fluorescent puncta (6×6 pixels = 760×760 nm) were obtained using Fiji software (‘Fiji Is Just ImageJ’, www.fiji.sc).

### Imaging of QD-BDNF and Alexa-BDNF Photostability

Photostability of QDs and Alexa dye under live neuronal imaging conditions was quantified as fluorescence intensity of a discrete QD and a region of Alexa_488_. Live NG neurons were treated with Ax_488_-BDNF (25 nM) and QD_625_-BDNF (200 pM) for 15 min, washed in PBS, and imaged with an epifluorescent microscope and camera (Zeiss Axiovert 200 M, PlanAPO 100x/1.4 oil objective, Andor iXon Ultra 897 EMCCD camera), at 18 fps for 10 minutes. An Optosplit II dual emission system (Cairn Research, Kent, UK) allowed simultaneous acquisition of two color channels (Alexa_488_ and QD_625_). Dual color channels were processed for total fluorescence intensity present in a 6×6 pixel (760×760 nm) square region for both QD and Alexa_488_ probes.

### Immunohistochemistry (EEA1, LAMP1)

Following QD-BDNF incubation (5 min) and fixation, cells were processed for immunohistochemical staining as described before [36]. Primary antibodies: anti-EEA1 (Abcam, Cat# ab2900, 1∶500), anti-LAMP1 (Abcam, Cat# ab24170, 1∶500). Secondary antibodies: donkey anti-rabbit-Ax_488_ (Life Technologies; Cat# A21206, 5 µg/ml) or goat anti-rabbit IgG-QD_605_ (Life Technologies; Cat# Q11402MP, 400 pM) for endosomal markers.

### Imaging of QD-BDNF probes in fixed neurons

Neurons were imaged with two inverted epifluorescent microscope and camera systems using Micro-Manager software (v1.4.13) [49]: 1) a Zeiss Observer, 63x/1.4 oil objective, Andor Luca EMCCD camera (Andor Technology, South Windsor, CT) equipped with a MJC001 2-x microscopy joystick console (ThorLabs, Sterling, VA) or 2) a Zeiss Axiovert 200 M, PlanAPO 100x/1.4 oil objective, Andor iXon Ultra 897 EMCCD camera equipped with a MS-2000 flat top XZY automated stage (Applied Scientific Instrumentation, Eugene, OR). One pixel equaled 127 nm. Fluorescence for Alexa Fluor488 was detected using 480 nm excitation and 535 nm emission wavelengths (Chroma Technologies Corp, Bellows Falls, VT). QD fluorescence was detected using filter cubes for QD_655_ and QD_625_ (Excitation 434 nm/Emission 620 nm and Excitation 434 nm/655 nm respectively; Semrock, Rochester, NY). In all experiments, cells were imaged over the total height of the cells by acquiring *z*-stacks (*z*-step = 300 nm).

### Imaging of QD-BDNF probes in live neurons

Live neuronal imaging was performed using an inverted epifluorescent microscope and camera (Zeiss Axiovert 200 M, PlanAPO 100x/1.4 oil objective, Andor iXon Ultra 897 EMCCD camera). Coverslips with neurons were placed in a chamber (Quorum Technologies, Guelph, Canada) and maintained at a constant temperature of 37°C during the live imaging using a heating inset (model P S1), a heating ring for the 100× objective, and a temperature control unit (Temp Module S1, PeCon GmbH, Germany). Following pulse QD-BDNF stimulation (1–2 min) and washout (1 min), movies were obtained by acquiring images of neurons at a fixed z plane (around mid-height of the neuronal soma or focused on neuronal processes), at frame acquisition times of 9 and 18 f/s, as dependent on selection of image size, for durations of 5–10 minutes. Trajectory analysis of QD-BDNF probes was performed on subsets of movies containing phenomena of interest in these acquired data sets.

### Determination of membrane vs. intracellular QD-BDNF complexes

Image analysis was performed to determine if QD-BDNF complexes were on the membrane or in the cytoplasm. Cellular image stacks of QD-BDNF and WGA-labeled membrane fluorescence were deconvolved (AutoQuant X2, MediaCybernetics, Rockville, MD). We developed a customized Matlab software (Mathworks, Natick, MA), ‘Cell Analyzer’, that performs super-resolution detection of fluorescently-labeled membrane location using a combination of phase congruency measurements [50] and threshold-based segmentation methods. This program localizes QD position by: 1) identifying candidate QDs in each image plane, 2) localizing the position with a sub-pixel accurate method (x-y resolution = 50 nm) based on radial symmetry [51], and 3) linking QDs from each *z*-plane and using the linking criteria that requires a QD at the same XY pixel to be present in at least 3 planes to account for blinking behavior of QDs, and 4) calculating the *z* position from its mean *z* position. 3D visualizations of QD and membrane positions and QD-BDNF and WGA-membrane merged images were generated respectively using Amira (VSG, Burlington, MA) and Fiji (‘Fiji Is Just ImageJ’, www.fiji.sc).

### Single particle QD-BDNF tracking

Single particle QD tracking was performed using custom developed software implemented in MATLAB: data was pre-processed by subtracting the camera offset and dividing by a gain factor to convert image data from raw output to Poisson distributed ‘counts’ as previously described [52], [53]. Areas in each image were identified as possible candidates for single particle fitting using a difference of Gaussian filtering step as described in [54] and each candidate area was fit using the maximum likelihood method described in [53]. Fits that were above a threshold intensity and matched the expected point spread function shape (log-likelihood ratio test described in [54]) were connected into trajectories. Trajectory connection was performed using a modification of the cost matrix approach [55]. Trajectories were visually inspected to verify absence of connection errors before further analyses. Channel alignment between the WGA image and the QD image was performed by selecting equal size regions from each channel and using the function *findshift* provided by the DipImage library (http://www.diplib.org/dipimage, [56]) to align the sum projection of each image stack. This yielded an offset of one channel relative to the other, after which small final adjustments were made by visually inspecting the color overlay of the two images. Trajectories were superimposed upon the sum image of the WGA according to the computed particle positions obtained by the tracking software discussed above.

### Dynamic analysis of QD-BDNF Trajectories

#### Speed Analysis

Smooth paths representing the particle trajectories were found by either (1) a second order polynomial fit to the x and y data or (2) making a spline fit to the x-position *versus* time and the y-position *versus* time. The number of segments used to make the spline fit was adjusted to give a smooth fit without truncating features. The position along the path for each time point was calculated by finding the closest point of the path for each particle position. Speed was calculated by fitting a first order polynomial to sections of the path-position versus time values. Positive values of speed indicate movement along the path in the direction away from the start position. *MSD Analysis*: Mean square displacement *versus* time interval was calculated from individual trajectories and fit to either a free diffusion or corralled diffusion model as described in [57].

## Results

### High levels of QD-BDNF-TrkB binding specificity in neurons

**Figure 1:**
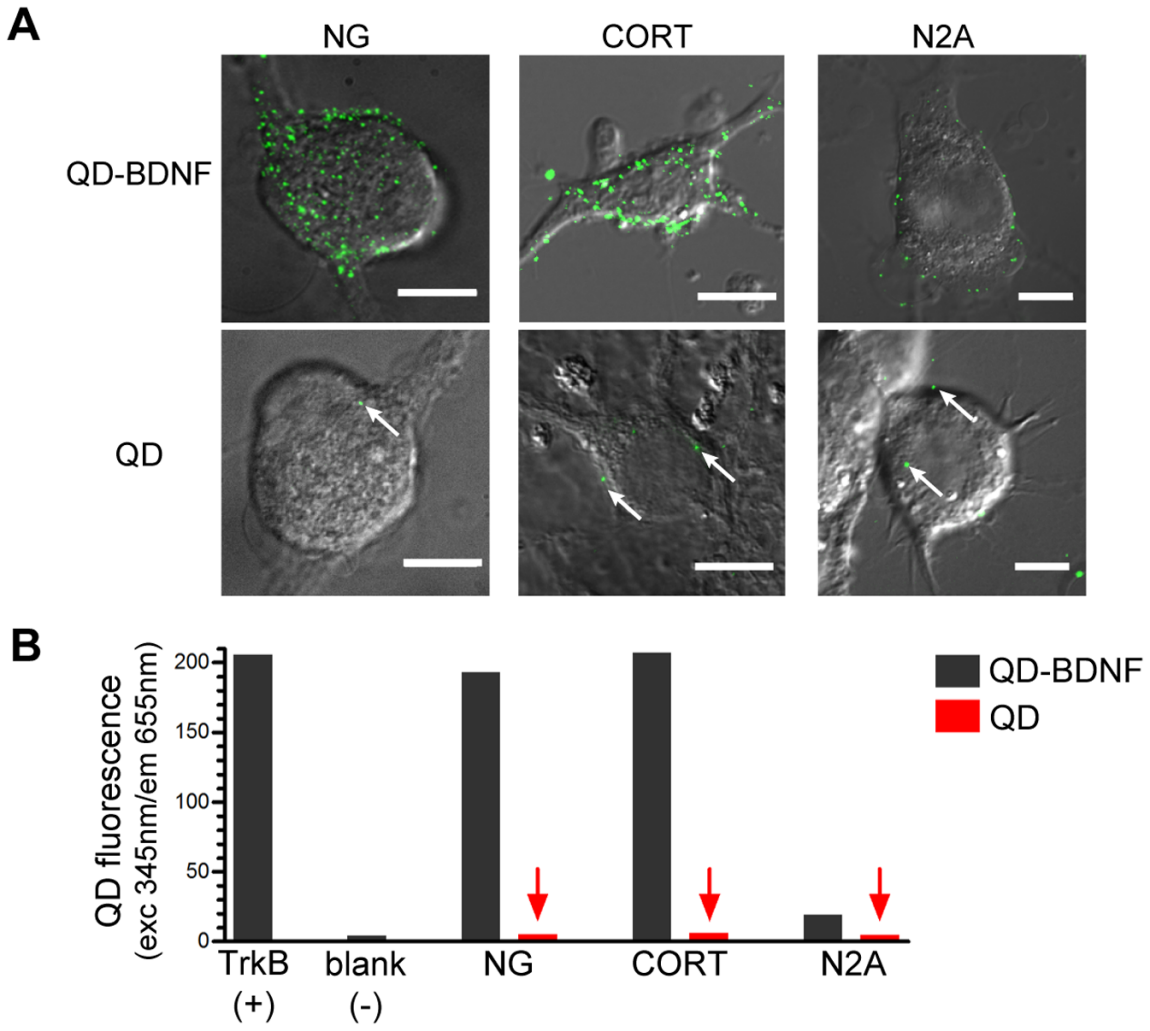
QD-BNDF binds with high molecular specificity to TrkB in live neurons. (A) Single cell assays show QD-BDNF probes (400 pM) bind preferentially to TrkB-expressing nodose (NG) and cortical (CORT) neurons vs. non-TrkB expressing control N2A neural cell lines. Control streptavidin-QD (400 pM) treatment typically showed non-specific binding levels of 0–2 QD/cell (white arrows; see text for more details). Collapsed *z*-stack micrographs (total cell height at 22–25 µm for NG, 5–8 µm for CORT and N2A). Scale bars: 10 µm. (B) Population assays by QD fluorescence measurement in neuronal lysates. Neurons treated with either QD-BDNF or streptavidin-QD (400 pM; black bars, red bars/arrows respectively), washed, and lysed. Positive control  =  TrkB receptor, negative control  =  lysis buffer blank.

Due to the single particle nature of QD studies, QD-BDNF probes must exhibit a high level of molecular specificity and it is necessary to confirm that we are studying QD-tagged BDNF complexes and not other non-specifically QD-bound protein. First, we determined and found that biotinylation of BDNF as well as the binding of streptavidin-QDs to biotinylated-BDNF did not reduce BDNF binding affinity to TrkB receptors by *in vitro* plate assay (Fig S1). We then assayed QD-BDNF binding specificity in live TrkB-expressing nodose ganglion (NG) sensory and cortical neurons [58], [59]. Overlay of DIC and QD fluorescence *z*-stacks over the entire cell height illustrates that QD-BDNF discrete fluorescent puncta were present at notably higher levels in NG and cortical neurons, compared to control (non-TrkB expressing) neuronal N2A cell lines (Fig 1A). Importantly, discrete counts of control streptavidin-QDs (400 pM) exhibited extremely low non-specific QD binding (83% of neurons had no QDs, 17% had 1–2 QDs; n = 100 cells). Similarly, population assays measuring QD-fluorescence in neuronal lysates also showed that QD-BDNF bound at significantly higher levels to NG and cortical neurons than control N2A cells (9.91 and 10.63 times higher than control N2A cells, respectively, Fig 1B, dark bars). Control streptavidin-QD treatment resulted in almost no binding (Fig 1B, red bars/arrows). Our QD fluorescence detection was reliable in these neuronal lysate assays as TrkB receptor-coated ELISA wells showed strong QD-BDNF fluorescence whereas negative blank buffer control showed no QD-BDNF fluorescence (Fig 1B). Altogether, these results indicate that QD-BDNF binds to TrkB in live neurons with a very high degree of molecular specificity.

### QD-BDNF activates downstream TrkB signaling pathways

**Figure 2:**
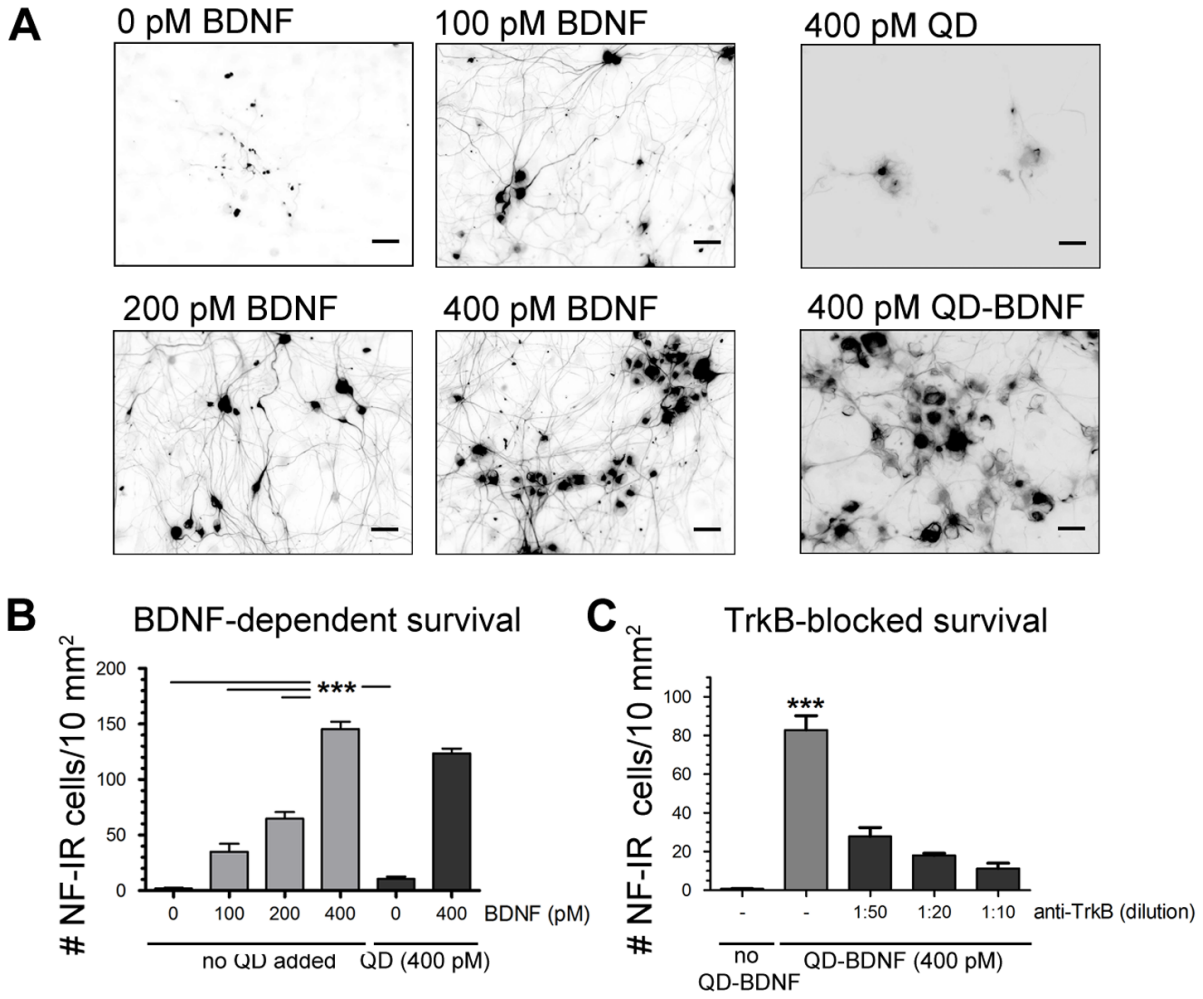
QD-BDNF probes activate TrkB signaling and induce neuronal survival. (A) Embryonic (E) 16.5 NG neurons incubated with QD-BDNF probes showed survival rates equivalent to unmodified BDNF (400 pM), while incubations with streptavidin-QDs did not. Neurons incubated with unmodified BDNF, streptavidin-QD and QD-BDNF (72 hr) are identified by anti-neurofilament (NF68/180) antibodies. (B) Quantitation of results shown in (A). Neuronal survival induced by QD-BDNF was indistinguishable from unmodified BDNF (ANOVA, p<0.0001, Dunnett's post-hoc test, n = 4). Density of neurofilament (NF68/180) immunoreactive (IR) cells increased as a function of BDNF and QD-BDNF concentration. (C) QD-BDNF-dependent survival is TrkB specific as pre-block with anti-TrkB receptors and subsequent QD-BDNF treatment decreased NG neurons survival (ANOVA, p<0.0001, Dunnett's post-hoc test, n = 5). Scale bar: 50 µm.

We determined whether, following the high molecular specificity of QD-BDNF-TrkB binding that we observe in live neurons, QD-BDNF-TrkB complexes induced downstream TrkB signaling. BDNF-TrkB signaling is essential for the survival of embryonic (E16.5) NG neurons [43], [60]. Increasing concentrations of unmodified BDNF (0–400 pM) produces higher neuronal survival and neurite outgrowth (Fig 2A). This well-established BDNF effect on NG neuron survival is mimicked by QD-BDNF. As expected, streptavidin-QD alone (400 pM, negative control) did not result in NG neuron survival (Fig 2A). Counts of cells immunoreactive for the pan-neuronal marker Neurofilament 68/160 (Fig 2B) showed that neuronal survival was significantly higher at the BDNF concentration of 400 pM for both, unmodified and QD-BDNF (Fig 2B). This QD-BDNF survival effect was specifically TrkB-mediated, as pre-incubation with TrkB function blocking antibodies prior to BDNF-QD exposure resulted in significantly decreased neuron survival in E16.5 NG cultures. Moreover, the survival decreased with increasing concentrations of anti-TrkB pre-treatment (Fig 2C). Altogether, these results show that QD-BDNF specifically binds and activates TrkB downstream signaling that directly supports neuronal survival.

### Localization of single QD-BDNF complexes

**Figure 3:**
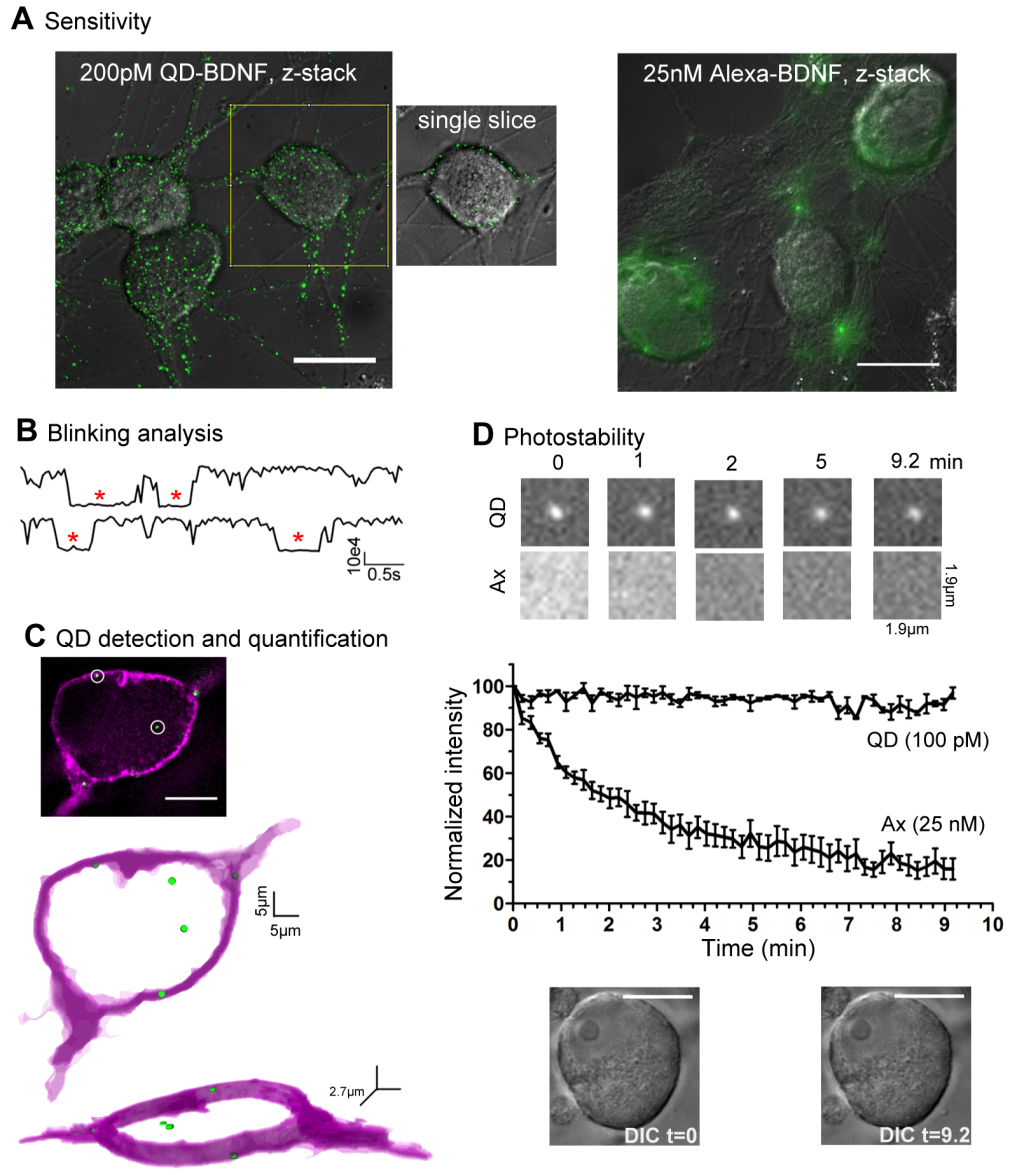
Photophysical properties of QD-BDNF-tagged TrkB receptors in live neurons. (A) Sensitive detection of discrete QD-BDNF complexes in NG neurons (200 pM, left panel) compared to diffuse TrkB labeling with Ax_488_-BDNF (25 nM, right panel).Images are collapsed *z*-stacks (total cell height, 21–22 µm). QD-BDNFs are also detectable in single *z*-slice slices (yellow inset). (B) QD-BDNF complexes are detected inside single neuronal somata as single QDs. Representative fluorescent blinking profiles from two QD-BDNFs inside a neuron show square pulses of single ‘on-off’ QD blinking (red asterisks). *x*-axis  =  intensity, *y*-axis  =  time. (C) Highly-resolved spatial detection of QD-BDNFs accurately determines membrane vs. cytoplasmic location of QD-BDNF complexes in neurons. 3D models of location of single QD-BDNF complexes (green) with respect to Ax_488_-WGA-labelled membrane (magenta) are computationally processed from raw fluorescence data (inset, 2.7 µm thick *z*-stack projection taken at neuronal mid-section). (D) QD-BDNF complexes within neuronal somata can be tracked for extended time durations. Single QD-BDNF vs. diffuse Ax_488_-BDNF fuorescence inside a neuron over time (top). Average intensity as a function of excitation duration show quantitative comparison of extended fluorescence stability of QD_625_-BDNF vs. Ax_488_-BDNF (n = 10, middle). Under these extended recording sessions, corresponding DIC images of a representative neuron before and after 10 min of fluorescence excitation shows maintained morphological integrity (bottom). Scale bars: 20 µm (A), 10 µm (C, D).

Addition of QD-BDNF probes (25–400 pM) to live neurons resulted in TrkB binding and the subsequent internalization of BDNF-TrkB complexes, that was readily detectable in single z-slices taken from NG neurons as discrete fluorescent puncta localized to the soma and processes (Fig 3A, left image). In comparison, staining of live neurons with Ax_488_-BDNF (25 pM to 25 nM) was found to be undetectable in single z-slices, and was only observed as diffuse staining in z-stack projections at concentrations x125 higher than the QD-BDNF concentration (Fig 3A, right image). The QD-BDNF concentration range of 25–400 pM also allows us to visualize single discrete QD-BDNF complexes at a concentration of QD-BDNF that produces physiologically relevant neuronal survival (Fig 2). QD-BDNF probes deposited on glass coverslips indicated that a significant majority were single QDs. Similarly, discrete QD-BDNF puncta inside neurons showed characteristic on-off square pulses or ‘blinking behavior’ that are indicative of single QDs (Fig 3B, 84.4%, n = 90). Thus, we can detect QD-BDNF complexes with single QD sensitivity, allowing localization at high spatial resolution of single QD-BDNF complexes with respect to fluorescent-labeled membrane (Fig 3C) that is valuable for assessing the BDNF-TrkB regulation in single cells (see next section).

An important property of the live tracking of QD-BDNF complexes is the capability to maintain QD-BDNF tracking over long durations needed to view time-dependent events. Depending on the QD coating, QD fluorescence can be susceptible to pH and salts present in the local cellular microenvironments [61]–[64], which has not been well examined for QD-tagged ligand-receptor complexes. Here, we optimized parameters including neutral density filters, vitamin C and HEPES buffer to control imaging conditions and the neuronal environment in order to track QD-BDNF complexes at sampling rates with maintained fluorescence lasting for as long as 10 minutes (Fig 3D, top trace in graph). The long-lasting QD-BDNF photostability contrasted that of Alexa-BDNF. In the same live neuronal preparations in which we optimized for Alexa-BDNF detection and could visualize Alexa-BDNF diffuse intensity (25 nM, Fig 3A), we found that Alexa-BDNF photobleached by 50% within 2 min. Under these fluorescence conditions, high-magnification bright field images of the same neuron taken before and after imaging showed no obvious signs of blebbing or unusual granularity, suggesting that neurons maintained their health (Fig 3D, bottom images).

All together, the QD-BDNF probe characterizations show that QD-BDNF binds with high-specificity to TrkB and induces TrkB-mediated signaling in live neurons. Furthermore, single QD-BDNF complexes can be detected for extended periods of time within neuronal soma, under live imaging conditions in which neurons continue to appear healthy.

### Time course of BDNF-TrkB internalization and trafficking

**Figure 4:**
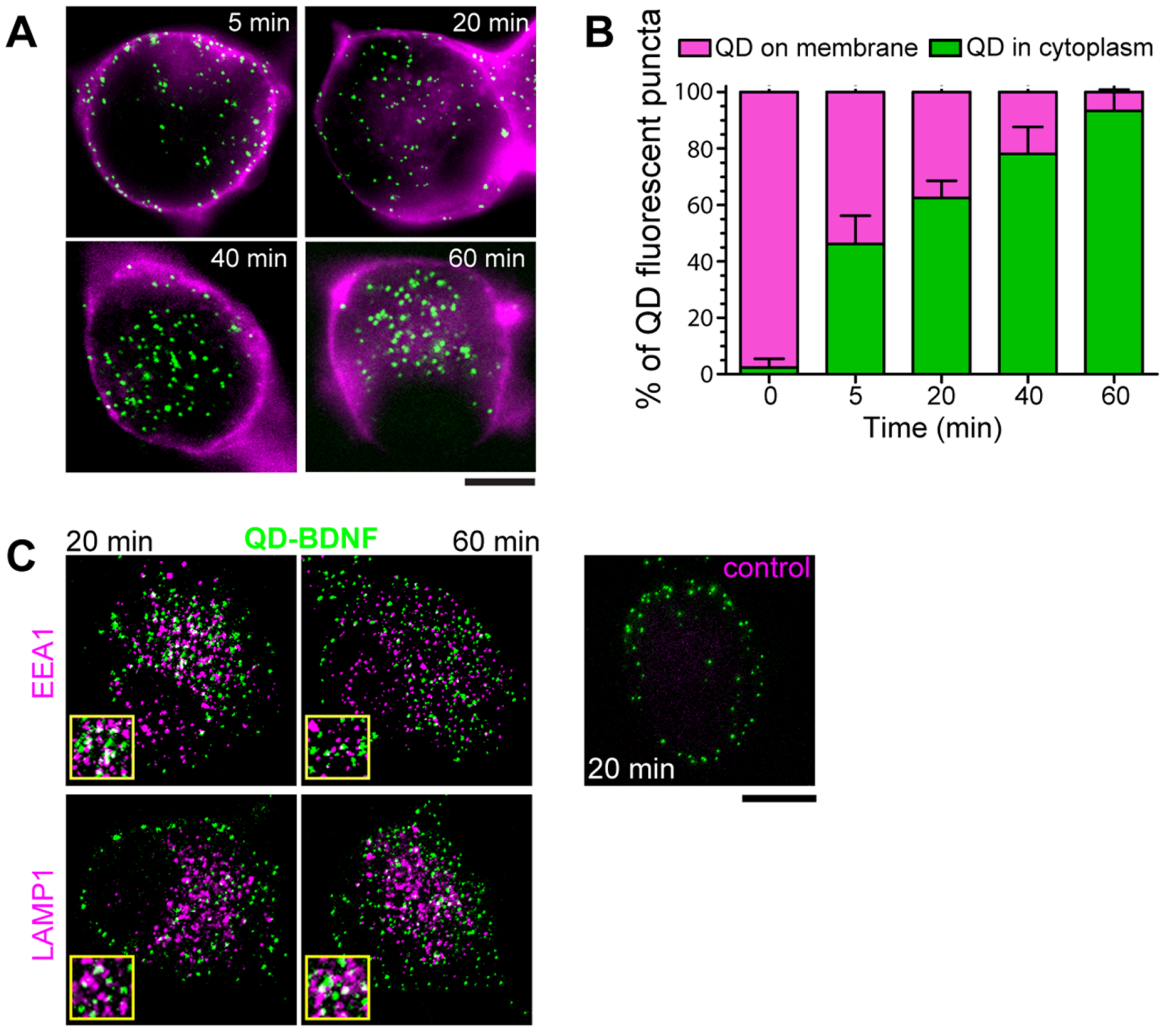
Time course of internalization of QD-BDNF-TrkB complexes. (A) QD-BDNF-TrkB complexes are visualized as fluorescent puncta (green) on the plasma membrane and within NG neurons. *Z*-stack projections (neuronal mid-section, 2.7 µm thick). WGA-Ax_488_ labeled plasma membrane (magenta). (B) Quantitative localization of single QD-BDNF complexes (green) in neuronal somata vs. plasma membrane (magenta) following QD-BDNF stimulation (200 pM, 5 min pulse). Each bar is the average of n = 8–10 cells; ∼30–50 QDs/cell for each time point. (C) Endosomal co-localization of QD-BDNF complexes (green) with anti-EEA1, and anti-LAMP1antibodies (Ax_488_, magenta) following a 5 min pulse of QD-BDNF (200 pM, green) at t = 20 and 60 min. Yellow insets show magnified co-localization (white). Control shown at topmost right is same as BDNF-QD + endosomal antibody labeling conditions except with omission of primary antibody. WGA-Ax_488_ labeled plasma membrane (cyan). Collapsed *z*-stack projections of neuronal mid-section (2.7 µm thick). Scale bars  = 10 µm.

The dynamic regulation of plasma membrane residing receptors is important for controlling the strength and time duration of downstream signaling. We applied QD-BDNF probes to study the internalization of BDNF-activated TrkB receptors at the level of single BDNF-TrkB complexes. Images of Ax_488_-WGA membrane-labeled NG neurons treated with a pulse of QD-BDNF (5 min, 200 pM) were fixed at subsequent time points (t = 5 min to 1 h). At early time points (t = 5 min), a majority of QD-BDNF-TrkB complexes were located near the plasma membrane, whereas at t = 60 min, the majority were located within neuronal somata (Fig 4A). Quantitative localization of single QD-BDNF complexes shows internalization reaching 60%, 80% and 90% at t = 20, 40 and 60 min, respectively (Fig 4B). The percentages of single internalized BDNF-TrkB complexes are likely higher at later time points (t = 40 and 60 min), as single QD-BDNF fluorescent puncta start to appear larger and brighter, and likely represent the combined merging of earlier endosomal contents into later trafficking endosomes. These data indicate that the overall internalization dynamics of BDNF-TrkB in the neuronal soma is a gradual process; at t = 20 min over a half of BDNF-TrkB complexes were within neuronal soma, and a few QD-BDNF fluorescent puncta were still present on the plasma membrane at t = 60 min (Fig 4A, 4B).

The endosomal compartments within which QD-BDNF-TrkB complexes were trafficked were determined by co-localization of QD-BDNF with early and degradative endosomal marker antibodies in NG neurons. Figure 4C shows co-localization of QD-BDNF with the EEA1 early endosomal marker and LAMP1 lysosomal marker at mid and later time points (t = 20 and 60 min) following QD-BDNF stimulation. QD-BDNF complexes co-localized with the EEA1 endosomes at t = 20 min, but not at t = 60 min. Moreover, QD-BDNF complexes co-localized with LAMP1 at t = 60 min, but not at t = 20 min (Fig 4C). These endosomal co-localization data show that QD-BDNF complexes are sorted into early endosomes and trafficked to degradative pathways, as it is consistent with data for BDNF-TrkB and other NGF-TrkA growth factor sorting into early and degradative endosomes [4], [65]–[67].

### Real time dynamics of BDNF complexes

We recorded the trajectories of individual QD-BDNF complexes in the soma of live sensory NG neurons. By recording for long time durations, we could observe diverse types of QD-BDNF intracellular trafficking events. All trajectories shown below are segments of recordings that were acquired within t = 3–12 min following a 1 min pulse of QD-BDNF stimulation and correspond within the time frame of our QD-BDNF endosomal co-localization and internalization studies (t = 5–20 min, Fig 4).

#### Dynamics of BDNF complexes at the plasma membrane and neural processes

**Figure 5:**
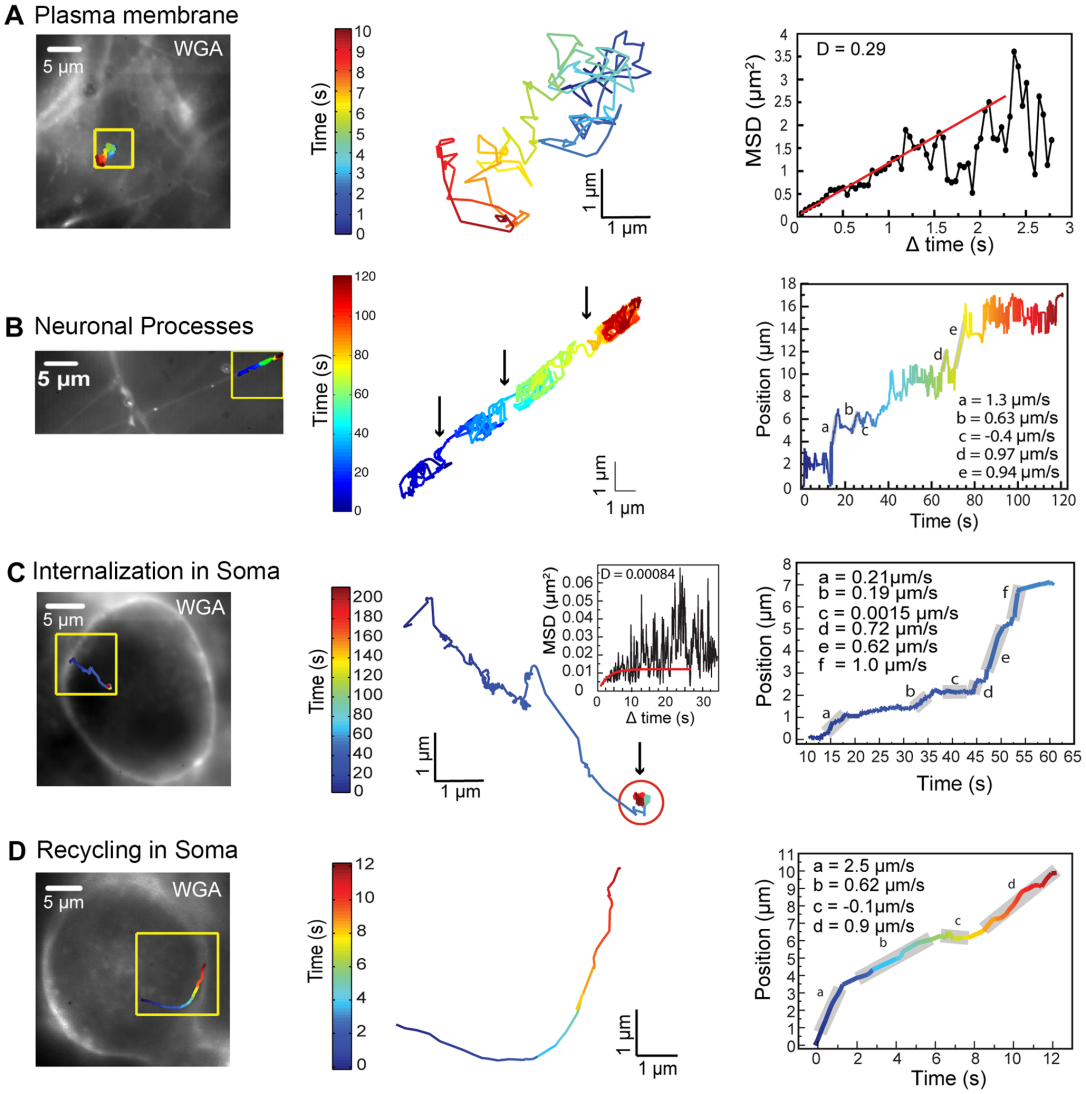
QD-BDNF diffusion dynamics at the plasma membrane, directed transport at neuronal processes, and internalization and recycling in the neuronal soma. Left panels: QD-BDNF trajectories (colored line) in relation to plasma membrane (left images) and magnified (yellow inset, right images). Middle panels: QD-BDNF colored trajectories in detail. Right panels: MSD or QD-BDNF position plots as a function of time, colored lines correspond to QD-BDNF trajectories in left panels, and gray bars represent speeds. (A) Trajectory and MSD plot for a QD-BDNF complex undergoing Brownian diffusion at the plasma membrane. (B) Trajectory and position plots of a QD-BDNF complex undergoing a mixture of diffusion and rapid transport along a neuronal process. Black arrows point to segments along the colored trajectory where rapid motor transport occurs; corresponding speeds are shown in adjacent position plots. (C)Trajectory and position plots of a QD-BDNF complex undergoing endocytosis that consists of phases of rapid transport as well as a long period of confined motion (red circle, MSD plot). (D) Trajectory and position plot showing a QD-BDNF complex recycling to the plasma membrane by rapid transport along a curvilinear path from deep within a NG neuron.

Following QD-BDNF treatment, QD-BDNF complexes could be seen at fluorescently labeled neuronal plasma membranes proximal to the cover glass. QD-BDNF complexes exhibited rapid diffusive motion that is characteristic and expected of receptor dynamics at the membrane (Fig 5A, left and middle panels; Movie S1). Mean square displacement (MSD) analysis of QD-BDNF trajectories confirmed the presence of Brownian motion with diffusion coefficient of D = 0.29 µm^2^/s (Fig 5A, right panel). This diffusion coefficient is relatively rapid compared to the rates of 0.01–0.1 µm^2^/s found in other neuronal receptors [68].

In neuronal processes, QD-BDNF complexes showed exploratory diffusive behavior that was interspersed with phases of linear transport that produced a net linear displacement along the process (Fig 5B left and middle panel). Linear transport had speeds of 0.63-1.3 µm/s (Fig 5B right panel) that are characteristic of active motor transport and similar to the motion of growth factor complexes in anterograde and retrograde directions along neural processes [8], [37], [39], [69].

#### Internalization dynamics of BDNF complexes

Individual QD-BDNF complexes could be observed in real time undergoing internalization into the neuronal soma (Fig 5C, left panel; Movie S2). Internalization events occurred as early as t = 3 min following a 1-min pulse of QD-BDNF but were also observed over the entire duration of recording (t = 3–12 min). By following the motion of individual QD-BDNF complexes for long durations of several minutes, it was apparent that QD-BDNF complexes exhibit a heterogeneous mixture of dynamic motions following internalization. Figure 5C and Movie S2 show a representative QD-BDNF complex undergoing an initial phase of rapid motion toward the cell center, followed by a phase of confined motion (Fig 5C, left and middle panels). Close examination indicated that the initial phase of rapid, directed motion was composed of a series of linear displacements with speeds ranging from 0.2–1.0 µm/s; these linear displacements were at times separated by short pauses (Fig 5C, right). The ‘stop-and-go’ nature of this rapid, directed motion is characteristic of active motor transport along cytoskeletal tracks [70], [71] and is similar to microtubule-based motion as observed for NGF-TrkA and other growth factors in neuronal processes [39], [41]. A notable feature of intracellular QD-BDNF trafficking was the occurrence of phases of apparent immobility or confined motion which could last from several seconds to minutes (Fig 5C, middle panel, red circle; Movie S2). MSD analysis indicated that confined QD-BDNF complexes were largely immobile (Fig 5C, middle panel, inset). Such phases of time-extended immobility not only followed rapid transport upon internalization of QD-BDNF complexes, but also were a commonly observed phenomenon of intracellular, post-endocytic trafficking (see intracellular trafficking below).

#### Recycling dynamics of BDNF complexes

The recycling of activated growth factor-Trk receptor complexes serves to sustain signaling by the rapid return of available receptors to the plasma membrane [25], [67]. Evidence supports that BDNF-TrkB complexes undergo recycling [15], [72] but the dynamics of this process have not been directly observed. By recording for extended time durations, we captured recycling events of QD-BDNF complexes moving from the cytoplasm to the plasma membrane (Fig 5D, left panel; Movie S3). BDNF recycling dynamics were comprised of a series of rapid, linear displacements at speeds ranging 0.62–2.5 µm/s that were interspersed with short pauses (Fig 5D, right panel), indicating that movement to the plasma membrane is mediated largely by rapid cytoskeletal transport. Interestingly, the recycling of QD-BDNF complexes back to the plasma membrane can originate from deep within the neuronal soma and traverse lengthy, curvilinear paths that span several microns, indicating that BDNF complexes do not necessarily rapidly recycle within endosomes proximal to the plasma membrane. We did not observe immediate release of QD-BDNF complexes into the extracellular medium upon return at the plasma membrane; the recycled QD-BDNF complex appears to remain at the plasma membrane (Movie S3).

Intracellular trafficking dynamics of BDNF complexes in the neuronal somata. Examination of the intracellular dynamics of BDNF complexes within the neuronal soma show that BDNF complexes undergo a wide diversity of trafficking events that are not synchronized in time, follow no particular sequence, and occur throughout the t = 3–12 minute duration following QD-BDNF treatment.

**Figure 6:**
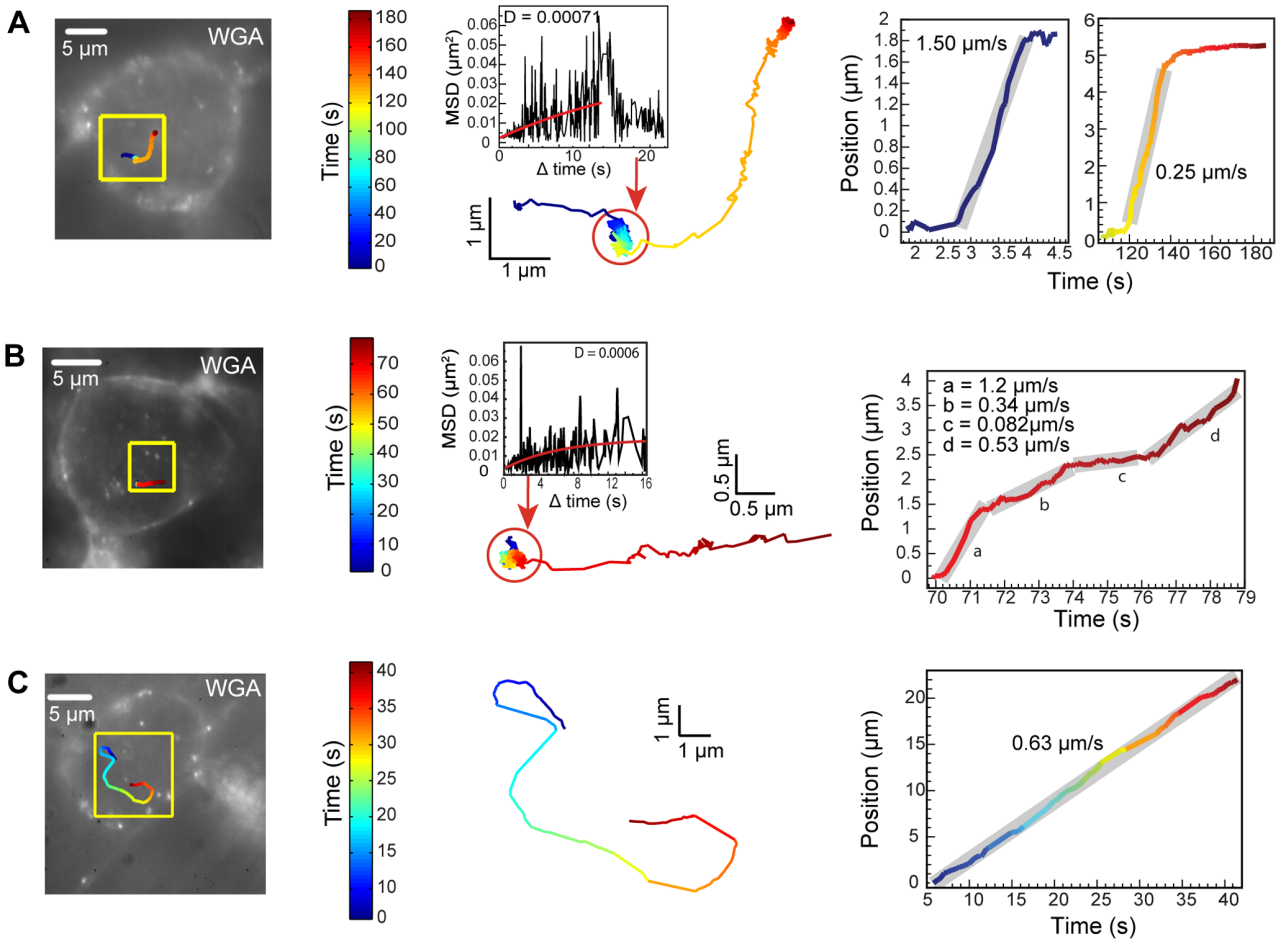
Intracellular trafficking dynamics of QD-BDNF complexes in the neuronal soma. Left panels: QD-BDNF trajectories (colored line) in relation to plasma membrane (left images) and magnified (yellow inset, right images). Middle panels: QD-BDNF colored trajectories in detail. Confined motion is circled in red (with corresponding MSD plots). Right panels: QD-BDNF position plots as a function of time, colored lines correspond to QD-BDNF trajectories in left panels, and gray bars represent average speeds. (A) QD-BDNF complexes undergoing intracellular trafficking that consists of phases of directed transport (blue curve in position plot), followed by confined motion (2 minutes), and a slower directed transport (yellow curve in position plot). MSD shows confined phase has a slow component (red circle). (B) QD-BDNF endosome trafficking that consists of an extended phase of confined motion (∼60 s; MSD plot, red circle), followed by rapid transport. (C) QD-BDNF endosome trafficking at a constant speed (35 s) along a curvilinear path (20 µm) within the neuron.

Intracellularly trafficked BDNF complexes exhibited widely heterogeneous dynamics. Phases of rapid, directed motion and immobility were intermixed. For example, during the course of several minutes of trafficking, QD-BDNF complexes experienced rapid, directed motion (1.5 µm/s) for tens of seconds, followed by sustained phases of apparent immobility lasting approximately 2 min, which was then followed again by rapid, directed motion (0.25 µm/s), lasting tens of seconds before encountering another phase of immobility (Fig 6A and Movie S4). Figure 6B shows a QD-BDNF complex that experienced a long period of immobility (70 sec) that was then followed by rapid, directed motion consisting of stop-and-go, with linear displacement speeds of 0.34–1.2 µm/s. Such sustained periods of confinement are surprising long in duration and, to our knowledge, have not been reported before. MSD analysis (Fig 6A and 6B, insets in middle panels) indicated that the QD-BDNF is largely confined. It is unlikely that the QD-BDNF complex is ‘stuck’ because rapid transport occurred following long durations of immobility (Fig 6A and 6B). These confinement periods could be observed soon following QD-BDNF stimulation (t-3–12 min), suggesting that the QD-BDNF complex is likely to be degraded in lysosomes which occurs approximately around t = 60 min following QD-BDNF stimulation (Fig 4C).

Unlike movement of QD-BDNF complexes along neuronal processes (Fig 5B), QD-BDNF complexes did not show a net-directional movement toward any particular destination in the neuronal soma (e.g. toward the cell center). Some QD-BDNF complexes appeared to be moving toward the center of the cell (Fig 6A) while others meandering along curvilinear trajectories throughout the neuronal soma (Fig 6C, Movie S5). The latter, QD-BDNF complexes could be found moving very rapidly without pause for surprisingly long durations along such meandering trajectories. Figure 6C and Movie S5 show an example QD-BDNF complex that moved at a constant speed for an extended period of time (35 s) over a curvilinear path of 22 µm in length. In summary, intracellular transport in the neuronal soma is characterized by an incredibly rich heterogeneity of intracellular dynamics that are neither synchronous nor follow an apparent net movement toward any particular subcellular destination. The heterogeneity of intracellular trafficking dynamics include expected rapid stop-and-go active transport as well as novel and surprising observations of extended durations of rapid transport, and immobility in the neuronal soma (Fig 6).

## Discussion and Conclusions

QD-BDNF probes do not interfere with BDNF-TrkB binding affinity and, in live neurons, bind with high molecular specificity to TrkB receptors, initiating downstream TrkB signaling. By optimizing live neuronal imaging conditions, it is possible to track single QD-BDNF complexes for extended durations inside neurons. This capability enables observations of the BDNF complexes undergoing diverse internalization, recycling, and intracellular trafficking events throughout the neuronal soma. BDNF complexes exhibit a wide variety of dynamics, including sustained rapid transport, stop-and-go transport, and confinement. These dynamic events were not clearly synchronized in time and could be observed over a wide duration of time (t = 3–12 min) following pulsed QD-BDNF treatment (1 min). Unlike the linearly-directed trafficking found in neuronal processes or the nuclear-directed trafficking of other intracellular complexes [73], [74], QD-BDNF complexes did not show a net directional gain toward any particular subcellular target destination. Instead BDNF complexes could be found meandering over surprisingly long durations (several minutes) and over long distances (up to tens of microns) throughout the neuronal soma. These new data, which are direct molecular measurements made within the live cell environment, paint a more complex picture of ligand-receptor trafficking dynamics that suggests a need for expansion of simplified models of nuclear-directed ligand-receptor signaling in the neuronal soma [1], [5], [7], [75]. This work also makes new contributions to the emerging development and application of probes for the study of intracellular trafficking dynamics of ligand-receptor complexes and other cargo within cells [36], [39], [42], [64], [70], [76]. QD-ligand probes are poised to not only dissect the intracellular molecular mechanisms underlying the intricate intracellular dynamics we report here, for normal as well as deviant ligand-receptor trafficking that is associated with many neurological disorders [11], [14], [28], [29], but also to provoke new understanding of the role of spatiotemporal dynamics in shaping cell signaling.

Comparison of our live BDNF trafficking studies to other studies, shows similar features in rapid, directed motion. However, we also report novel trafficking dynamics that show prolonged durations in both, motion and immobility, which, to our knowledge, have not been previously observed. The rapid, directed motion dynamics described here, in which BDNF complexes experience stop-and-go linear displacements over a range of speeds (0.2–1.2 µm/s) is similar to that described for microtubule and other cytoskeletal-based endosomal transport. The short stops seen between these linear displacements (Fig 5C, 6A, 6B) are similar to those described for molecular motor switching [9], [71], [77]–[79]. Interestingly, however, are the differences seen in the extended duration (several minutes) in which BDNF complexes stay immobile in the intracellular neuronal soma. In addition to these sustained phases of immobility, also novel is the occurrence of sustained phases of rapid, directed motion (up to 40 s) in which the QD-BDNF complex travels at a constant speed for tens of microns throughout the soma. These two trafficking phenomena may not have been previously observed due to the lack of technical tools to measure molecular scale dynamic events for long periods of time in live cell preparations. The underlying mechanism producing immobility could be caused by BDNF complexes switching and/or stalling to explore all possible microtubule tracks with which to engage in a crowded cell environment [79], [80], as well as the merging of multiple endosomes into larger multivesicular bodies. Immobility is unlikely due to slowed trafficking in degradative endosomes as we observe immobile phases soon following QD-BDNF exposure and prior to the BDNF-lysosomal trafficking that occurs about 60 min following QD-BDNF treatment (Fig 4C). Future investigation is required to elucidate the underlying molecular mechanisms that give rise to each of these intricate dynamics and to determine if such characteristics are impaired in animal models of neurological conditions.

With an average cell body diameter of 20–25 µm, a QD-BDNF complex could directly travel to the nucleus of the NG neuron in about 10–15 seconds (at average speed of 1 µm/s). Instead, we observe trafficking events where the BDNF complex moves within the cell for durations of minutes, with no clear cellular destination. Current models of endosomal signaling propose that growth factor-Trk and G protein-coupled receptor (GPCR) signaling complexes undergo linear, nuclear-directed transport inside cells, signaling within the cytoplasm as they move en route toward the nucleus [1], [2], [81]. The meandering BDNF intracellular dynamics described here differ however from such models and suggest that other types of mechanisms contribute to forming cellular signaling in neuronal soma. Undoubtedly, a valuable future area of study lies in determining the relationship between the path trajectories that QD-BDNF complexes traverse and the location of focused QD-BDNF spatial application in live neurons and its relation to effectors of downstream MAPK/PI3K pathway activation.

Our QD-BDNF studies which focus on the neuronal soma rather than the plasma membrane or axonal processes reveal new BDNF trafficking dynamics that contribute to an integrated understanding of the spatiotemporal dynamics of growth factor signaling. The detailed and comprehensive characterization of the QD-BDNF probes greatly validates them for studies where high specific binding to, and downstream activation of, TrkB receptors is important. Also, in addition to our evidence showing typical endosomal sorting, the wide diversity of trafficking dynamics phenomena we observed support the value and validity of the QD-BDNF probe, which holds great promise for use as reliable intracellular reporters of BDNF and other ligand-receptor dynamics within the soma of neurons and other cells.

## Supporting Information

Figure S1 **QD-BDNF binding to TrkB not impeded by BDNF biotinylation and streptavidin-QD conjugation.** (A) Confirmation of BDNF biotinylation by ELISA assay using anti-BDNF capture antibodies and avidin-HRP detection. Biotin-BDNF (solid line) increased HRP activity compared to unmodified BDNF (dashed line) indicating a successful biotinylation. (B) Neither biotinylation nor streptavidin-QDs affects BDNF binding to TrkB receptors. Biotin interference assay (left *y*-axis): ELISA plates coated with TrkB receptors and incubated with biotin-BDNF (solid black line) or unmodified BDNF (dashed black line), followed by anti-BDNF antibodies, and HRP/TMB detection. Streptavidin-QD interference assay (right *y*-axis): ELISA plates coated with TrkB receptors and incubated with QD-biotin-BDNF (solid gray line) or a control mixture of streptavidin-QDs + unmodified BDNF (dashed gray line). QD fluorescence was measured at 655 nm.
(TIF)

Movie S1 QD-BDNF complex undergoing Brownian motion at the neuronal plasma membrane. Movie shows Brownian motion of a QD-BDNF-TrkB complex on the plasma membrane (red trace). Video acquired at a fixed *z* (mid-height of the cell body) at 9 f/s. Fluorescent QD (green) and Alexa-WGA labeled plasma membrane channels acquired simultaneously.
(MP4)

Movie S2 QD-BDNF complex undergoing endocytosis into the neuronal soma. Movie shows internalization of a QD-BDNF-TrkB complex from the plasma membrane to the cytoplasm of the neurons (red trace). Video was acquired at a fixed z (mid-height of the cell body) at 9 f/s. Fluorescent QD (green) and Alexa-WGA labeled plasma membrane channels acquired simultaneously.
(MP4)

Movie S3 QD-BDNF complex undergoing recycling from within the neuronal soma to the plasma membrane. Movie shows recycling of a QD-BDNF-TrkB complex from the cytoplasm to the plasma membrane of the neurons (red trace). Video was acquired at a fixed *z* (mid-height of the cell body), at 9 f/s. Fluorescent QD (green) and Alexa-WGA labeled plasma membrane channels acquired simultaneously.
(MP4)

Movie S4 QD-BDNF complex exhibits intermixed phases of rapid transport and confinement in the neuronal soma. Movie shows cytoplasmic trafficking of a QD-BDNF-TrkB complex (white dots) that is characterized by rapid stop-go-stop-go movement and long durations (∼2 min) of the confined motion (red trace). Video was acquired at a fixed z (mid-height of the cell body), at 18 f/s.
(MP4)

Movie S5 QD-BDNF complex showing rapid transport along a lengthy, curvilinear trajectory in the neuronal soma. Movie shows cytoplasmic trafficking of a QD-BDNF-TrkB complex (white dots), characterized by a long (22 µm) curvilinear motion (red trace). Video was acquired at a fixed z (mid-height of the cell soma), at 18 f/s.
(MP4)
