# Supplementary figures and images for: Heterogeneous Intracellular Trafficking Dynamics of Brain-Derived Neurotrophic Factor Complexes in the Neuronal Soma Revealed by Single Quantum Dot Tracking
Source: PLoS One. 2014 Apr 14;9(4):e95113. doi: 10.1371/journal.pone.0095113 (PMC3986401; doi:10.1371/journal.pone.0095113)

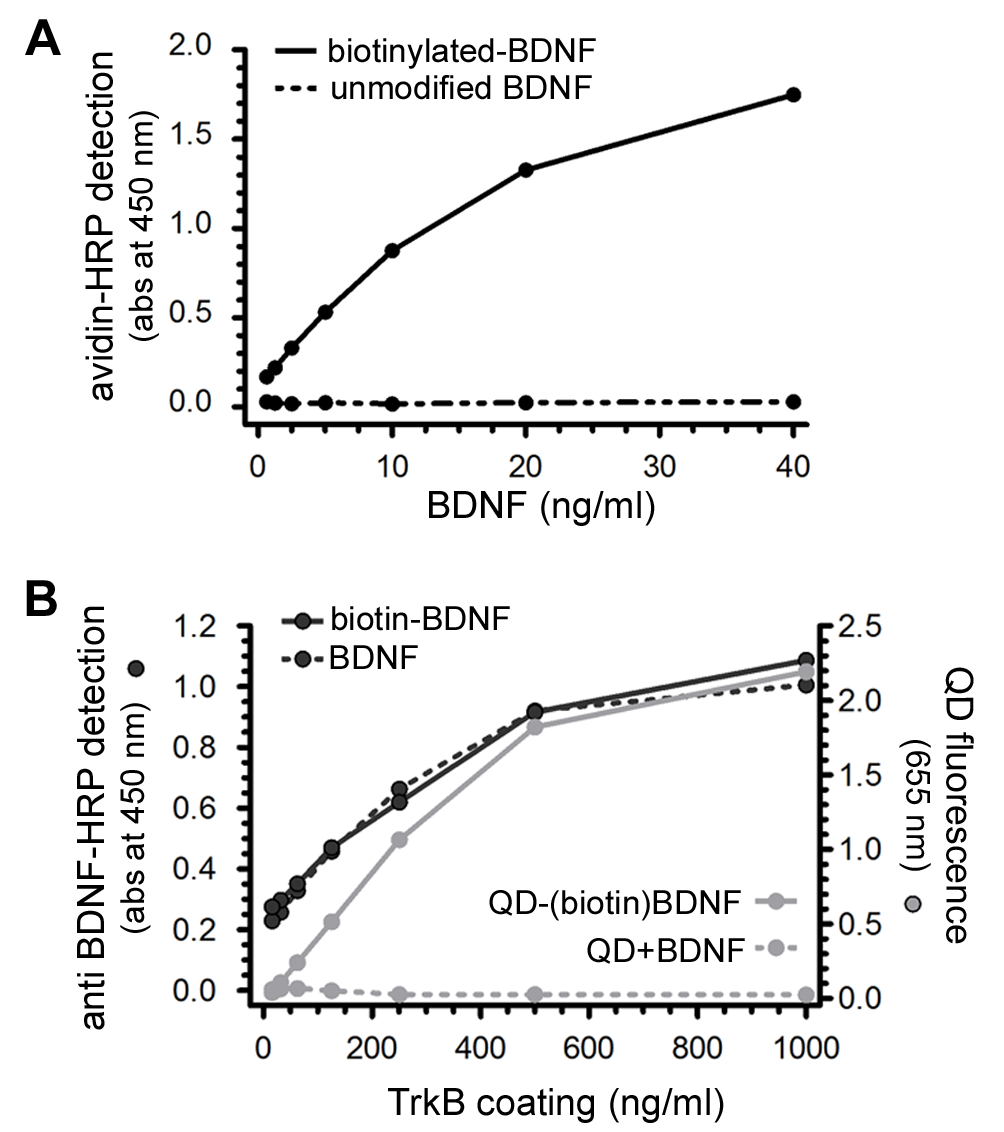

Supplement: Figure S1 — QD-BDNF binding to TrkB not impeded by BDNF biotinylation and streptavidin-QD conjugation. (A) Confirmation of BDNF biotinylation by ELISA assay using anti-BDNF capture antibodies and avidin-HRP detection. Biotin-BDNF (solid line) increased HRP activity compared to unmodified BDNF (dashed line) indicating a successful biotinylation. (B) Neither biotinylation nor streptavidin-QDs affects BDNF binding to TrkB receptors. Biotin interference assay (left y-axis): ELISA plates coated with TrkB receptors and incubated with biotin-BDNF (solid black line) or unmodified BDNF (dashed black line), followed by anti-BDNF antibodies, and HRP/TMB detection. Streptavidin-QD interference assay (right y-axis): ELISA plates coated with TrkB receptors and incubated with QD-biotin-BDNF (solid gray line) or a control mixture of streptavidin-QDs + unmodified BDNF (dashed gray line). QD fluorescence was measured at 655 nm. (TIF) [file pone.0095113.s001.tif]
